# Mass‐Standardised Differential Antibody Binding to a Spectrum of SARS‐CoV‐2 Variant Spike Proteins: Wuhan, Alpha, Beta, Gamma, Delta, Omicron BA.1, BA.4/5, BA.2.75 and BA.2.12.1 Variants—Antibody Immunity Endotypes

**DOI:** 10.1111/imm.70083

**Published:** 2025-12-09

**Authors:** Philip H. James‐Pemberton, Shivali Kohli, Jordan Twynham, Aaron C. Westlake, Alex Antill, Rouslan V. Olkhov, Andrew M. Shaw

**Affiliations:** ^1^ Biosciences, University of Exeter Exeter UK; ^2^ Attomarker Ltd Exeter UK

**Keywords:** antibodies, vaccination, viral

## Abstract

A fully mass‐standardised quantitative comparative analysis of the differential antibody binding to spike variant proteins to SARS‐CoV‐2 has been performed for the variants: Wuhan, Alpha, Beta, Gamma, Delta and the Omicron variants BA.1, BA.2.12.1, BA.2.75, BA.4 and BA.5. Evolution of immunity through five patient cohorts (*n* = 148 in total) was studied including pre‐pandemic, first infection, first vaccine, second vaccine and triple‐vaccinated cohorts. A population of immunity endotypes has been observed and is classified against a recovery antibody threshold, with concentrations below this threshold being regarded as a ‘dropout’: U(+) showing protection to all variants; U(±) with single, double, triple and further dropout endotypes; and U(−) with all variant concentrations being under the threshold. The U(+) incidence rises significantly following multiple rounds of vaccination reaching an (*n* = 41) incidence of 54% (95% CI 39%–68%) suggesting between half and three‐quarters of the population have universal variant vaccine antibody protection. The U(+) epitopes are targeted preferentially to the S1 region. U(±), with at least one dropout, has an incidence of 42% (95% CI 28%–57%), an immunity gap. Further, a U(−) sub‐cohort of the population up to 13% does not make antibodies above the threshold and may not have a sterilising serum leading to persistent virus and a risk of Long COVID.

## Graphical abstract

Fully quantitative, mass‐standardised, antibody responses to ten SARS‐CoV‐2 variants show significant variation in immunity profiles or endotypes. The incidence of the complete response to all variants is significantly improved after three vaccinations. Up to 13% of profiles are very low concentrations for all variants and point to risks in viral persistence such as long COVID.

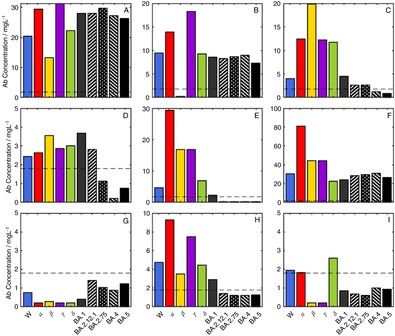

## 1 Introduction

The response to immunisation is personalised with antibody production based on affinity‐matured clones selected against epitopes on the spike proteins of the SARS‐CoV‐2 pathogen [1]. Vaccination and infection, ideally, should mature antibodies and T cells to conserved epitopes that are resistant to mutation. However, the choice of the affinity‐matured epitope may depend on the first exposure to the antigen, which is then retained in B cells and produced for all subsequent exposures to the antigen—the concept of original antigenic sin [2, 3, 4]. If the epitope is not conserved during mutation of the viral protein target, the antibody response may become significantly impaired. The rapid mutations observed [5] in SARS‐CoV‐2 made the initial vaccines redundant, well‐targeted to the Wuhan variant but quickly evaded by later variants. Bivalent mRNA vaccines produce spike proteins for both the Wuhan and Omicron variants, BA.5 [6] (Pfizer) or BA.1 [7] (Moderna) and more recently, FDA approval of monovalent vaccines targeting the KP.3 strain. Some studies with different vaccine combinations [8, 9] have shown improvement in the resulting neutralising antibody protection.

Evaluation of vaccine efficacy needs to keep pace with the evolution of the virus and hence harmonised [10] correlates of protection (CoP) and correlates of clearance (CoC) are required to bridge the variants or be re‐derived. Ideally, the CoPs and CoCs should be replaced with a mass‐standardised mechanism of protection and clearance which is not dependent on the variants, that point to accurate characterisation of the serum antibody profile [11]. By comparison, CoPs are likely variant specific [12]: fivefold variation in Receptor Binding Domain (RBD)‐ACE2 binding affinities has been observed for different variants [13, 14]; and more interestingly increased viral load in later variants [15]. Neutralising antibody (NAb) assays have shown a decline in antibody titres with variant [16], reflecting the conserved epitope map in the NAbs produced in the patient response triggered by vaccine, infection or complex exposure pattern.

In this paper, we explore the variation of the mass‐standardised (NIST standard antibody [11]) of individual patient response to the set of SARS‐CoV‐2 variants: Wuhan, Alpha, Beta, Gamma, Delta and Omicron BA.1, BA.4, BA.5, BA.2.75 and BA.2.12.1. Comparison with the mechanistic threshold derived previously [11] of 1.8 mg/L (0.2–3.4 mg/L (95% CI)) was used to assess patient immunotype. Patient samples were screened from five cohorts with varying infection and vaccine profiles (*n* = 148 in total): pre‐pandemic (controls); the Wuhan wave pre‐vaccine; and then single, fully vaccinated, and boosted cohorts for select combinations of vaccines, boosters, and infections. A classification of immunity endotype is derived from the observation of different immune responses for protection and clearance. A Fischer exact test was used to assess the significance of the incidence of different endotypes between the cohorts, and a Marascuilo procedure to identify between which cohorts. Wilson 95% confidence limits were determined for each of the cohort endotype incidences.

## 2 Methods and Materials

### 2.1 Methods

#### 2.1.1 Biophotonic Multiplexed Immuno‐Kinetic Assay

A biophotonic platform, such as localised particle plasmon or continuous gold sensor, fundamentally consists of effective‐mass sensors responding to the changes in refractive index in the plasmon field once it has been excited. The localised particle plasmon technique used here utilises an immuno‐kinetic assay which has been described in detail elsewhere in a SARS‐CoV‐2 antibody sensing application [11, 17]. Briefly, gold nanoparticles are printed into an array of 170 spots which are then individually functionalised with the SARS‐CoV‐2 spike proteins from each of the variants described in Table S1, alongside control spots of recombinant Human Serum Albumin for variation in illumination, temperature and non‐specific binding. A protein A/G control channel measures total IgG and is used to assess antibody integrity in the calibration reagents.

The integrity of the protein samples on the surface was tested using a panel of antibodies raised to RBD, S1, S2 or whole S protein, detailed in Table S1. An anti‐S2 antibody (40590‐D001) was chosen for calibration as the corresponding epitope is present on all variants. The concentration of the antibody was calibrated against the NIST antibody RM8671, NISTmAb, a recombinant humanised IgG1κ with a known sequence [18] to assure monomeric purity of the antibody. High‐control and low‐control samples were made using the 40590‐D001 antibody, and these controls were then used to quantify results from human samples in units of mg/L.

All 96 samples were collected within one antibody half‐life (60–200 days) [19], 23 samples were collected above 60 days (Table 1). The median days since the double‐vaccination were 43 days (26–121 days lower and upper quartile) and for the triple‐vaccinated cohort the median was 32 days (20–63 days lower and upper quartile). A technique‐independent endotype classification is based on the diagnostic accuracy threshold derived from four patient cohorts: recovering from infection (*n* = 200 PCR(+) and 200 controls); percentile of two vaccine response distributions that are resistant to live viral challenge; and a set of circulating IgG positive standards [11]. The average of all thresholds is 1.8 mg/L (95% CI 0.2–3.4 mg/L) and a universal positive endotype, U(+), has the antibody concentrations for all variants above the 1.8 mg/L threshold. A single drop‐out has an antibody concentration for one variant below the threshold, for example, β(−), double drop‐out, two variants α(−) β(−) etc., for all variants and these are all labelled U(±); one or more variant antibody concentrations < 1.8 mg/L. U(−) has a concentration below threshold for all variants. A further classification of transient endotypes, UT, was made to allow for waning of antibody concentration with time; the antibody levels were doubled and UT(+), UT(±) and UT(−).

**TABLE 1:** Demographic data for samples from vaccinated individuals collected in the Attomarker clinic.

Vaccine	Total number of donors (*n* = 96)	Percentage male	Percentage female	Min. days since last dose	Max days since last dose	Mean days since last dose
Single vaccination						
ChAdOx1‐S (AZ)	9	44	56	17	71	52
BNT162b2 (Pfizer)	4	75	25	10	77	43
Double vaccination						
ChAdOx1‐S (AZ)	23	30	70	7	196	65
BNT162b2 (Pfizer)	17	47	53	6	297	83
Unknown	1	100	0	149	149	149
Triple vaccination						
ChAdOx1‐S (AZ) + Pfizer/Moderna	21	57	43	6	87	32
BNT162b2 (Pfizer) + Pfizer/Moderna	13	46	54	1	82	43
CX‐024414 (Moderna)	1	100	0	18	18	18
Novavax (cross‐over trial) + BNT162b2 (Pfizer)	1	0	100	29	29	29
Unknown	5	60	40	63	167	126
Unknown vaccination	1	—	—	—	—	—

### 2.2 Statistical Analysis

The incidence of endotypes within each cohort was tested for significant differences using first Fisher's Exact test extended to multiple variables, then a Marascuilo Procedure [20] to test each pair of cohorts in turn against a significance level of 0.05. Both methods adjust the significance input to account for the increasing Type I error risk stemming from multiple cohorts being analysed simultaneously. A full correlation analysis for all combinations of responses was also performed to assess whether responses to one variant correlated with or predicted the response to another variant: correlation coefficients and lines of best fit were derived.

### 2.3 Materials

Materials used throughout the course of the experiments were used as supplied by the manufacturer, without further purification. Sigma‐Aldrich supplied phosphate buffered saline (PBS) in tablet form (Sigma, P4417), phosphoric acid solution (85 ± 1 wt. % in water, Sigma, 345245) and Tween 20 (Sigma, P1379). Glycine (analytical grade, G/0800/48) was provided by Fisher Scientific. Assay running and dilution buffer was PBS with 0.005 v/v % Tween 20, and the regeneration buffer was 0.1 M phosphoric acid with 0.02 M glycine solution in deionised water.

The recombinant human antibody to the spike protein S2 subdomain was a chimeric monoclonal antibody (SinoBiological, 40590‐D001, Lot HA14AP2901). The antibody was raised against recombinant SARS‐CoV‐2/2019‐nCoV Spike S2 ECD protein (SinoBiological, 40590‐V08B). The panel of antibodies seen in Table S2 was used to screen antigens for relative integrity and epitope presentation.

NISTmAb, Humanised IgG1κ Monoclonal Antibody from National Institute of Standards and Technology (RM8671). The NISTmAb is a recombinant humanised IgG1κ with a known sequence [18] specific to the respiratory syncytial virus protein F (RSVF) [21]. The detection mixture consisted of a 200‐fold dilution of IG8044 R2 from Randox in assay running buffer.

Two sensor chip designs were printed; an Omicron focused array with recombinant human serum albumin (rHSA) from Sigma‐Aldrich (A9731), protein A/G (PAG) from ThermoFisher (21186), and six SARS‐CoV‐2 Spike Protein variants for the Wuhan and trimer proteins for Omicron BA.1, BA.2.12.1, BA.4, BA.5 strains from SinoBiological and BA.2.75 spike protein from Acro Biosystems. The other sensor chip design included earlier variants, with rHSA, PAG and six monomer Spike Protein variants: Wuhan, Alpha, Beta, Gamma, Delta and Omicron BA.1. The complete protein data can be found in Tables S1 and S2.

### 2.4 Patient Samples

#### 2.4.1 Commercial Samples

Serum samples were purchased from two suppliers (Biomex GmbH and AbBaltis). Seventeen pre‐pandemic (pre‐December 2019), PCR(−) human serum samples were purchased from AbBaltis. All were tested and found negative for STS, HbsAg, HIV1 Ag (or HIV PCR(NAT)), HIV1/2 antibody, HCV antibody, and HCV PCR(NAT) by FDA‐approved tests. Eleven positive samples were purchased from AbBaltis, which were all from PCR(+) individuals. No information was provided regarding the symptoms of the donors. Forty‐five percent of these samples were from female donors, and 55% were from male donors. The age of donors ranged from 19 to 81 years. No information on the time from infection to sample collection was given.

Samples purchased from Biomex (*n* = 26) were from PCR(+) individuals. All samples were YHLO Biotech SARS‐CoV‐2 IgG positive and Abbott SARS‐CoV‐2 IgG positive. A spectrum of patient symptoms from the following list was detailed for each sample: fever, limb pain, muscle pain, headache, shivers, catarrh, anosmia, ache when swallowing, diarrhoea, breathing difficulties, coughing, tiredness, sinusitis, pneumonia, sickness, lymph node swelling, pressure on chest, flu‐like symptoms, blood circulation problems, sweating, dizziness and hospitalisation. Time from infection to sample collection ranged from 27 to 91 days. 35% of these samples were from female donors and 65% were from male donors.

The samples were collected prior to June 2020; PCR(+) samples will result from infection by the SARS‐CoV‐2 strains circulating prior to this time, most similar to the Wuhan protein. These commercial samples, 38(+) and 16(−), were tested using the Spike Variant Array as a part of this study.

#### 2.4.2 Clinical Samples

Samples were collected from patients in partner clinics, all of whom provided informed consent for their anonymised data to be used in research to aid the pandemic response. The data from tests of 96 patient samples are included in this study: 13 had received one dose of either AstraZeneca (AZ) or Pfizer SARS‐CoV‐2 vaccine; 41 had received two doses of either AZ, Pfizer or Moderna SARS‐CoV‐2 vaccine at least 14 days prior to sample collection and testing by Attomarker; 41 further samples were from patients who had received a third vaccination. Full patient demographics are shown in Table 1. One sample had no vaccine history disclosed.

#### 2.4.3 Cohort Allocation

The samples were split into five cohorts for analysis based on their infection and vaccination characteristics:

- Pre‐Pandemic (*n* = 16): Samples collected before December 2019.
- Wuhan (+) (*n* = 36): Samples with PCR confirmed SARS‐CoV‐2 infection prior to June 2020.
- 1× Vaccine (*n* = 13): Samples where the donor had received 1 dose of a SARS‐CoV‐2 vaccine.
- 2× Vaccine (*n* = 41): Samples where the donor had received 2 doses of a SARS‐CoV‐2 vaccine.
- 3× Vaccine (*n* = 41): Samples where the donor had received 3 doses of a SARS‐CoV‐2 vaccine.

Further information about the specific vaccination history for the vaccinated samples can be found in Table 1. Data were initially analysed with different vaccine combinations as separate sub‐cohorts, but the data were then combined according to the number of vaccinations to allow for significant results to be attained from the small sample numbers.

#### 2.4.4 Ethical Approval

The use of the Attomarker clinical samples with the consent of the patients was approved by the Bioscience Research Ethics Committee, University of Exeter.

## 3 Results

**FIGURE 1:**
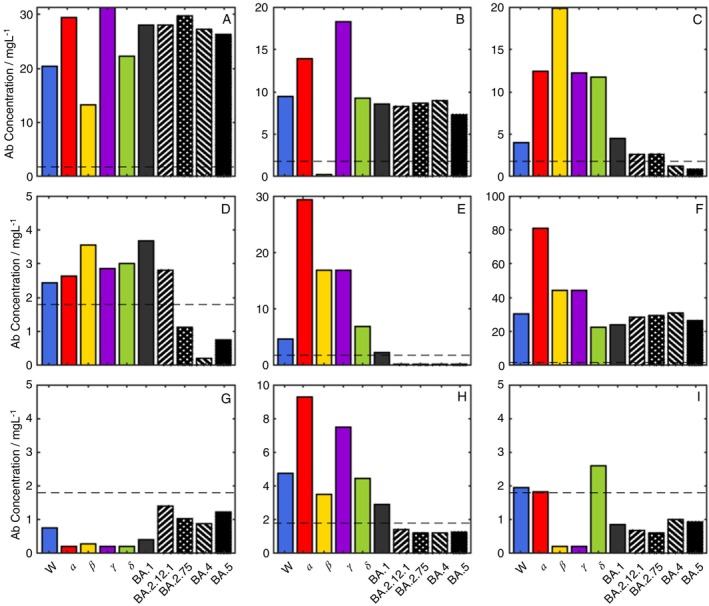
Immunity Endotypes—the dotted line is set at the 1.8 mg/L threshold: (A) a universal positive, U(+) [3× Pfizer vaccination]; (B) Single Dropout β(−) [3× Pfizer vaccination]; (C) Double Dropout BA.4(−) BA.5(−) [W(+) infection]; (D), Triple Dropout 2.75(−) BA.4(−) BA.5(−) [2× AZ]; (E) Quadruple Dropout BA.2.12(−) BA.2.75(−) BA.4(−) BA.5(−) [1× AZ]; (F) Super Responder [2× AZ—M]; (G) U(−) [2× AZ]; (H) UT() Quadruple dropout that becomes U(+) using half‐life rule [W(+)]; (I) U(±) Septuple dropout that changes to quintuple dropout using half‐life rule [W(+)] showing higher level of antibody epitope selection.

**FIGURE 2:**
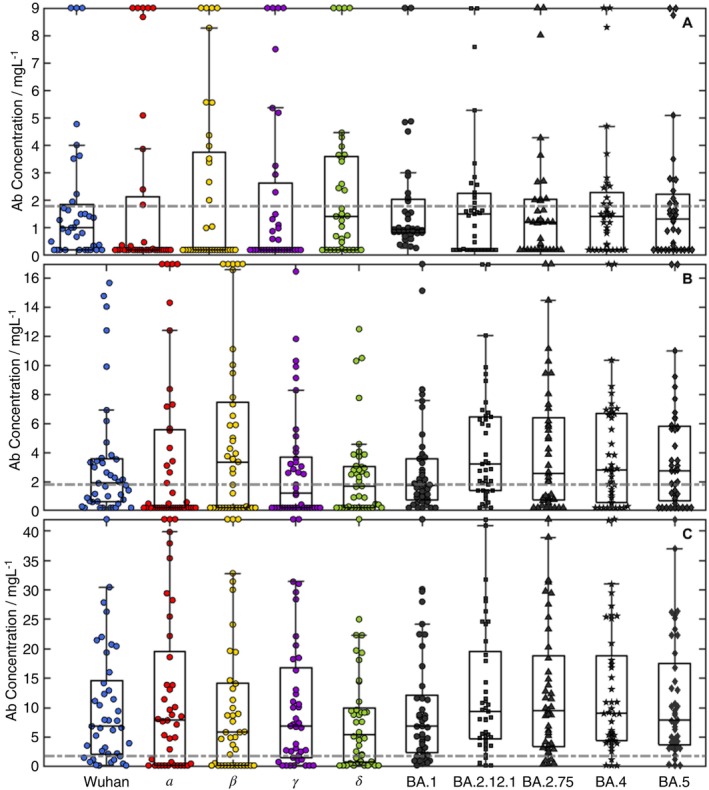
Bee swarm plots for three cohorts: (A) W(+); (B) double‐vaccinated patients; and (C) triple‐vaccinated patients. The boxes show upper and lower quartiles centred on the median, and the whiskers indicate the presence of outliers at 1.5× the interquartile range, with the whisker bar falling on the highest/lowest non‐outlier data point. Outliers are shown at y‐maximum and detailed in Table 1 (Values for the outliers on the top of the plot are shown in Table S7). The dotted grey line is drawn at 1.8 mg/L, the threshold used to separate a ‘dropout’ for the purposes of classification.

The pan‐variant integrity of a chimeric monoclonal antibody to the S2 region of the Spike protein was established by measuring the antibody binding to the spike protein panel, deriving a binding maximum within a Langmuir adsorption model (Table S3) as a quantitative measure of site density. The antibody was then used as the calibrant for the antibody binding spectrum, mass‐standardised against the NIST standard antibody. The antibody binding variant spectra and endotype classification of some patient responses are shown in Figure 1, with the population distributions for all samples shown in Figure 2 (and detailed in Table S6). The combined patient cohorts showed a range of immunity endotypes in response to the variant spectrum of SARS‐CoV‐2 spike proteins. The pre‐pandemic, negative control samples were U(−) = 81% (95 CI 57%–93%) spike antibody negative, with three patients showing ninefold dropouts suggesting immunity potentially due to a misclassification, a PCR false‐positive, previous alphacoronavirus infection or random antibody cross‐reactivity.

The variation in antibody spectra for the W(+) recovery, double‐vaccinated and triple‐vaccinated cohorts is significant (Figure 1) with the full endotype classification shown in Table 3 (and detailed in Table S9). The ideal antibody response to a universal epitope present in all variant spike proteins, U(+) (Figure 1A) was observed in 11% (95% CI 4%–25%) of the immunologically naïve Wuhan(+) patients, whilst 58% (95% CI 42%–73%) of patients presented with one or more dropouts, U(±). The most prevalent U(±) was a triple drop‐out (Figure 1D), β/γ/δ(−). The initial vaccination cohort has no U(+) endotypes, rising to 22% (95% CI 12%–37%) in the double‐vaccinated cohort and rises again in the triple‐vaccinated cohort to 67% (CI 50%–80%). The double‐vaccine groups for AZ or Pfizer offer different protection across the spectrum of variants with AZ showing U((1–8)−) dropouts dominated by U(5−) including the Omicron sub‐variants (Figure 1F–I). The Pfizer vaccine, by comparison, only has U (1–4) including a full mix of all variants. In the triple‐vaccination cohort, the higher‐order dropouts are nearly eliminated with no U (5) or greater endotypes. One patient with a triple vaccination also had an octuple drop‐out (Figure 1I), suggesting a lack of immunity across all variants and immunocompromised.

The variation in classification with time was considered by allowing for the variation of one half‐life and doubling all the antibody concentrations to produce a new UT classification. The proportions of each class in the cohorts do not change significantly, all falling within the 95% confidence limits.

The cohort distributions of the antibody spectra are shown in Figure 2. The pre‐vaccine infection cohort (Figure 2A), shows median antibody levels for Wuhan variants and small levels for Omicron subvariants. These median levels are increased in double and booster vaccination groups (Figure 2B,C). The interquartile range for Wuhan, natural infection cohort is low, with antibody levels varying from 0.2–1.8 mg/L, doubling in the double‐vaccinated cohort to 0.6–3.6 mg/L and rising further in the triple‐boosted cohort, 2–14 mg/L. The plots do not show the personalised detail of responses, particularly super‐ or low‐ responders; the lower outliers are clustered at the detection limit (200 ng/mL) and the upper outliers are grouped at the top of the figure (Table S7 shows the values for these patients). Of these 17 super‐responders, nine show a super‐response (beyond the 95th percentile of the population) to more than one variant and three show a full spectrum super‐response. The response to one variant can be used to predict the response of others; assuming a linear correlation, the *R* ^2^ (Table S5) and straight‐line coefficients are shown in Table S4.

### 3.1 Statistical Analysis

A Fisher Exact test was used to determine if any differences in Endotype (U(+)/U(−)/U(±)) proportions between the cohorts (Pre‐pandemic/W(+)/1×/2×/3× Vaccine) were significant, with a finding of *p* < 0.0001.

A Marascuilo Procedure [20] was run for each endotype across the five cohorts and found significant differences in the incidence rates of endotypes between cohorts (Table 2). The Marascuilo Procedure allows adjustment for the multiple cohorts being compared against a *χ* ^2^ distribution derived from the whole data set, allowing a significance level of *p* = 0.05 to be tested while adjusting for the increasing probability of Type I error, variation of *p* between different cohorts (Table S8). The significance level for each cohort comparison was used to construct (Table 2). Wilson 95% confidence limits were calculated for the proportions of each endotype in the cohorts. The analysis shows an increase in the proportion of samples with U(+) after three vaccinations for all cohorts. Although the improvements in drop‐out endotypes U(±) and U(−) do not improve outside of the uncertainty of the cohorts (Table 3).

**TABLE 2:** Significance tables showing whether the incidence rate of each endotype was significantly different between cohorts.

	Pre‐pandemic	Wuhan(+)	1× vaccine	2× vaccine
U(+)				
Pre‐pandemic	—			
Wuhan(+)	No	—		
1× vaccine	No	No	—	
2× vaccine	Yes	No	Yes	—
3× vaccine	Yes	Yes	Yes	Yes
U(−)				
Pre‐pandemic	—			
Wuhan(+)	Yes	—		
1× vaccine	Yes	No	—	
2× vaccine	Yes	No	No	—
3× vaccine	Yes	Yes	No	No
U(±)				
Pre‐pandemic	—			
Wuhan(+)	Yes	—		
1× vaccine	Yes	No	—	
2× vaccine	Yes	No	No	—
3× vaccine	No	No	No	No

*Note*: These were calculated using a Marascuillo procedure and a significance level of 0.05. The full table of values can be found in Table S8. The results are symmetric with respect to the cohorts being compared, so the table has not been completed above the diagonal.

**TABLE 3:** Summary Incidence of the endotype classifications in the five cohorts studied.

Endotype	Overall incidence (%) (*n* = 148)	Endotype incidence (%) by cohort														
		Pre‐pan (16)	W (+) (36)	1× vaccine (13)		2× vaccine (41)			3× vaccine (41)							Un (1)
				AZ (9)	Pf (4)	AZ (23)	Pf (17)	Un (1)	AZ‐AZ‐Pf (12)	AZ‐AZ‐M (9)	Pf‐Pf‐Pf (10)	Pf‐Pf‐M (3)	M‐M‐M (1)	Nx‐Pf (1)	Un (5)	
Summary (%)																
U(+)	26 (19–33)		11 (4–25)			26	18		58	89	40	33	100		60	100
Sub‐cohort				0 (0–28)		22 (12–37)			59 (43–72)							
U(−)	22 (16–30)	81 (54 ‐ 95)	31 (18–47)	22	50	8.7	12		8.3							
Sub‐cohort				31 (13–58)		10 (4–23)			2.4 (0.4–13)							
U(±) any dropout	52 (44–60)	19 (5–46)	58 (42–73)	78	50	65	71	100	33	11	60	67		100	40	
Sub‐cohort				69 (42–87)		68 (53–80)			39 (26–54)							
Dropout endotypes (±) (%)																
Single	2.7						5.9	100	8.3		10					
Double (−)	0.7		2.8													
Triple (−)	6.1					8.7	24				10			100	20	
Quadruple (−)	5.4		8.3	11		4.3	12		8.3							
Quintuple (−)	8.8		5.6	11		13	18			11.1	10	33			20	
6(−)—fold	6.8		14	11			5.9		8.3		10	33				
7(−)—fold	3.4		5.6			8.7					10					
8(−)—fold	5.4		8.3	11	25	8.7	5.9									
9(−)—fold	13	19	14	33	25	22			8.3		10					
UT (%)																
UT(+)	28		14 (6–30)			26	29	100	58	89	40	33	100		60	100
Sub‐cohort				0		29 (18–44)			59 (43–72)							
UT(−)	13	56 (33–77)	14 (6–30)	11	25	4.3	5.9		8.3							
Sub‐cohort				15 (4–42)		4.9 (1.3–16)			2.4 (0.4–13)							
UT(±) any dropout	59	44 (23–67)	72 (56–84)	89	75	70	65		33	11	60	67		100	40	
Sub‐cohort				85 (58–96)		66 (51–78)			39 (26–54)							

*Note*: Each incidence is presented as a percentage with the Wilson 95% confidence limits. The individual drop‐out classifications are detailed in Table S9.

**FIGURE 3:**
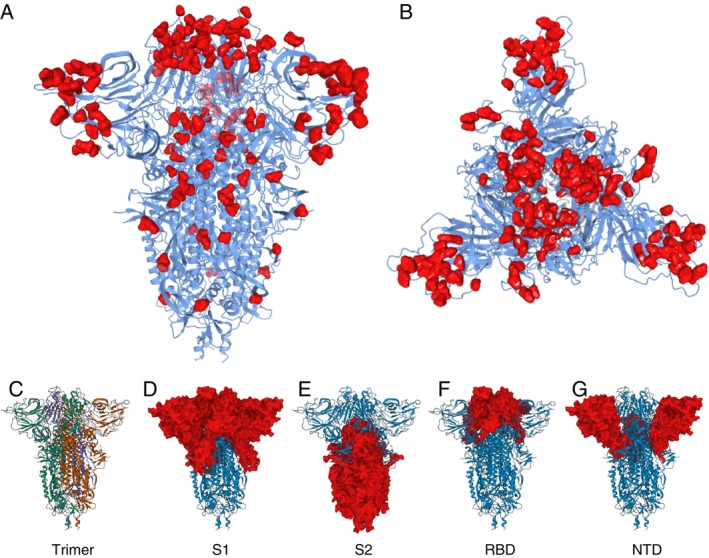
The superimposed Alpha, Beta, Gamma, Delta, BA.1, BA.2.12.1, BA.4/5 mutations of the SARS‐CoV‐2 Spike protein shown from (A) side‐on and (B) top‐down views. Also shown for context are (C) The spike trimer, with monomers differentiated, (D) S1 in red, (E) S2 in red, (F) Receptor Binding Domain (RBD) in red and (G) N‐terminal Domain (NTD) in red. Image created using the Swiss Institute of Bioinformatics Expasy tool [22].

The mutation of the spike protein during the evolution of the variants leads to a mutation map for the S1 and S2 regions (Figure 3), in particular the conserved epitopes that are associated with the U(+) endotype (Figure 3A,B). Further, smaller numbers of mutations are observed in the S2 region (Figure 3E), with a limited role as a neutralising antibody owing to its proximity to the viral particle membrane surface.

## 4 Discussion

Four independent patient cohorts were studied to explore the pan‐variant antibody response to the spike protein of SARS‐CoV‐2. The natural immune response from naïve patients exposed to the Wuhan escape variant is compared with single‐vaccinated, double‐vaccinated, and triple‐vaccinated cohorts and a further control, pre‐pandemic cohort. The vaccination cohorts were all targeted at the Wuhan spike protein, first‐generation vaccines corresponding to the circulating variant at the time. The antibody response spectra show significant differential antibody binding concentrations against the variant spike proteins, which would be expected to reflect variant infection protection, recovery and clearance. Critically, all measurements were mass‐standardised against NIST standard antibody [18] and fully quantitative, allowing direct comparison between variants. Endotype classification was determined against a single threshold of 1.8 mg/L (95% CI 0.2–3.4) mg/L measured previously [11] showing broadly U(+) antibody concentrations to all spike proteins above thresholds, U(±) with one or more concentrations below the threshold and U(−), all concentrations below the threshold (Figure 1).

Using the immunity endotype classification, it can be seen there is a significant endotype variation in the immunity of the patient cohorts, some of which may not be protective against some variants. The ideal antibody response produces a universal endotype, U(+), with antibodies binding to conserved epitopes present on the spike protein in all variants (Figure 3). There pre‐pandemic cohort has a U(−) incidence of 81% (CI 54%–95%) with three patients having positive responses to α variant (*n* = 2) and β variant (*n* = 1)—presumably arising from clones happening to be responsive to epitopes appearing on these spike variants. The U(+) incidence for this cohort is 0%. The W(+) naïve cohort (pre‐vaccination) has antibodies matured against the Wuhan spike protein during an infection and has a U(+) incidence of only 11% (95% CI 4%–25%) and there is no significant improvement in the first vaccine cohort (Table 2). The W(+) cohort maintains a high incidence of the U(−) endotype, 31% (95% CI 18%–47%). Most patients in the W(+) showed at least one gap to a variant in the spectrum, 58% (95% CI 42%–73%); the changes in U(−) and U(±) were significant changes from the Pre‐Pandemic profile.

The first vaccination cohort did not see a significant improvement in the incidence of the U(+) endotype relative to the Pre‐Pandemic or W(+) cohorts: 31% (95% CI 13%–58%) showing a U(−) endotype and 69% (95% CI 42%–87%) selecting epitopes with one or more dropouts; a non‐ideal vaccine response in line with the vaccination program of two doses to confer protection. The endotype distribution improves significantly with double vaccination and after three doses the U(+) endotype has an incidence of 59% (95% CI 43%–72%), significantly better than even the twofold vaccination cohort, with the rest of the cohort splitting broadly between U(−) and U(±). The antibody population levels (Figure 2) were reasonable across all variants, the population immunity for different variant waves Figure S1, was not well prepared for spike protein mutations although by the triple vaccination, the cohort had a U(+) incidence of 59% (95% CI 43%–72%). Repeat exposure to the same mRNA message produced significantly different immune endotype U(+) response incidence, breaking original antigenic sin [2, 3, 4].

The critical measure for population immunity is the incidence of U(+). In the single‐vaccination cohort the U(+) incidence was zero (Table 3). However, U(+) incidence in the double and triple vaccination cohorts rises: 22% (95% CI 12%–37%) and 59% (CI 43%–72%), respectively; both significantly larger. The vaccination cohorts may also have had asymptomatic infections reflecting a background of natural immunity and vaccine induce immunity throughout the time of collection in 2021. An anti‐nucleocapsid antibody assay would have been an option to test for viral exposure, but it has been shown to not conclusively rule out previous contact with the virus [23]. The rising U(+) incidence, however, points towards significant immunity evolution, B clone selection and maturation. The mRNA vaccine antibodies may be raised against any part of the spike protein, which is all included in the message and with a personalised glycosylation pattern [24], giving rise to a large U(±) endotype presentation, greatest for the double‐vaccinated cohort 68% (95% CI 53%–80%). The U(±) dropout improves close to significantly with the triple‐vaccination cohort falling to 39% (95% CI 26%–54%).

The robustness of the endotype classification depends on the initial quality of the threshold and its evolution in time. The threshold is derived from four cohorts (*n* = 400) including vaccine response distributions Pfizer and AZ vaccines, a diagnostic thresholds for patients recovering from natural Wuhan infection and circulating NIBSC standards [11]. The antibody concentrations leading to the endotype profiles will decrease over time, re‐upregulated during a subsequent challenge. The clone set may be the same leading to original antigenic sin [2, 3, 4] or refined further against the target. Antibody concentrations wane with a personalised half‐life reported to vary between 60–200 days for natural antibodies following infection [19]. The collection time of all patient samples (Table 1), is within 60 days of infection or vaccination. The time‐independence of the endotype classification was tested by doubling the antibody concentrations to produce UT(+), UT(−) and UT(±) classifications (Table 3). The result is the number of UT(−) is reduced in the vaccination cohort consequently increasing the UT(±) compared to U(±) from 69% (95% CI 42%–87%) to 85% (95% CI 58%–98%), a near‐significant change. There is no significant change in the other cohorts suggesting the U(+) and U(−) endotypes are reasonable representations of immunity profiles in the community. Significant up‐regulation of antibody production such as during acute infection (10‐fold antibody production) may change the endotype classification but it may be reasonably concluded that the endotype spectrum is a good estimate of antibody immunity.

The patient response to viral infection is moderated by many factors including Human Leucocyte Antigen (HLA) genes [25] conferring preferential viral response [26], suggesting a mechanism for asymptomatic SARS‐CoV‐2 infection [27], and prior T cell immunity targeting antibody development [28] and maturation [29]. Further, a pre‐configured paratope‐epitope bias postulated in the B cell receptor (BCR) binding and presentation has been reported [30], leading to rapid and targeted neutralising antibody production. The pre‐configured bias is not fully understood and there are clear vulnerabilities leading to sub‐populations with poor humoral responses to SARS‐CoV‐2. The bias may explain the prevalence of W(−) drop‐out in the cohorts, only improving with triple vaccination.

Structure–function and epitope stability on the target spike protein are essential to optimising vaccines against significant and rapid mutations: the Omicron subvariant mutations occurred in 70 days [31] while responses to update the vaccine were achieved in around 100 days [32]. A hemispherical region (comprising the RBD and NTD) at the top of the spike S1 region (Figure 3) is relevant to antibody protection [33] via both neutralisation and opsonisation. In addition, a second epitope region around the hinge would prevent the pre‐fusion‐fusion transition. By contrast, the RBD has acquired a significant number of mutations affecting infectivity and immune‐evasiveness of each strain, with 15 out of 222 amino acids changing [34], and of these nine occurring in the Receptor‐Binding Motif (RBM) [35], 14% of the total. Conversely, the NTD is relatively well conserved, with BA.1 only acquiring seven changes [36]. A kinetic epitope mapping study [37] based on clones from the universal endotype could be useful in the design of a universal ‘Hinge Vaccine’ and new monoclonals for immunotherapy.

Strikingly, the antibody endotypes are sensitive to S1 and not S2 regions of the spike protein (Figure 3). The U(+) incidence is 11% (4%–25%) in the naïve patient exposure to Wuhan, the first exposure leading to both cellular and humoral responses to the virus and full recovery. Similarly, in the vaccination cohorts where U(+) was rising but not an incidence of 100% despite the repeated presentation of the entire protein, S1 and S2 (Figure 3). Further, the gap in the antibody spectrum for the Beta variant from a triple Pfizer vaccination (Figure 1) is derived from a vaccine that presents whole Spike trimer consistently, even if there are modifications to RBD or S1 more widely. These observations point to a selection or maturation process that favours the prevention of disease or clearance of the virus post infection with maturation of antibodies paratopes in the S1, RBD and NTD regions. Consequently, the U(+) endotype points to antibodies reactive towards conserved epitopes in the regions of S1.

## 5 Conclusions

The mass‐standardised variant immunity spectrum reveals a set of immunity endotypes in each of the cohorts with a rising incidence of U(+), the universal conserved endotype response. The host response appears initially subject to a pre‐determined bias and then optimised to epitopes providing recovery, producing memory B cells and T cells with chosen epitope regions of the proteins, potentially unique to each host and leading to immunity imprinting [4, 38, 39]. The immunity imprint is not conserved in three cohorts studied here with the number of drop‐out endotypes falling with the occurrence of vaccination and infection. Indeed, the evolution away from drop‐out endotypes towards the U(+) universal response points to a universal vaccine or immunotherapy and a potential route to suppression of the virus. However, there are 39% of patients with U(±) and worse up to 10% with U(−), for which a good quantity of antibodies to the infecting variant is never present. The low concentration and potentially low affinity antibodies, define a non‐sterilising serum leaving patients less able to clear the virus and more vulnerable to post‐viral sequalae consistent with the persistent virus hypothesis for long COVID [40, 41, 42, 43]. It is widely recognised that this syndrome likely has multiple underlying mechanisms [44] and deficiencies in the humoral system could explain a subset of the disease.

## Funding

The work was funded by donations from the Exeter University Alumni during the pandemic, by Attomarker Ltd. that funded the PhD studentship for Philip James‐Pemberton at the University of Exeter and Attomarker Ltd. directly. Alex Antill was an undergraduate at the University of Exeter performing the work as part of his degree.

## Conflicts of Interest

Prof Shaw is the Founder, CEO and Director of Attomarker Ltd., a spin‐out company from his research group.

## Supporting information


**Table S1:** Variant spike proteins, manufacturer, cell line, modifications, length and mutations.
**Table S2:** Panel of antibodies used to screen spike protein integrity on the surface.
**Table S3:** Mean and standard deviation of *θ*_max_ values determined from the binding of aS2 antibody 40 590‐D001 to each protein channel. As this is the first time out—shall we include this?
**Table S4:** Table of line‐of‐best fit gradients, column = row × gradient + intercept. The intercept is the limit of detection.
**Table S5:** Table of *R* ^2^ values.
**Table S6:** Table of medians and quartiles, broken down by exposure cohort. All values are in mg L^−1^.
**Table S7:** Antibody concentrations for the Patients present in Figure 3 displayed on the upper limit of the figure.
**Table S8:** Full results from Marascuillo Procedure analysis of Cohorts. C1 = Pre‐Pandemic, C2 = Wuhan(+), C3 = 1× vaccine, C4 = 2× vaccine and C5 = 3× vaccine.
**Table S9:** Full endotype profile for the cohort detailing the dropout variants.
**Figure S1:** Prevalent SARS‐CoV‐2 variants in the UK November 2021–September 2022. (A) Estimated number of cases and (B) Variant share as % of genomes sequenced from a random sample.

## Data Availability

The data that support the findings of this study are available on request from the corresponding author. The data are not publicly available due to privacy or ethical restrictions.
