# Supplementary material for: Mass‐Standardised Differential Antibody Binding to a Spectrum of SARS‐CoV‐2 Variant Spike Proteins: Wuhan, Alpha, Beta, Gamma, Delta, Omicron BA.1, BA.4/5, BA.2.75 and BA.2.12.1 Variants—Antibody Immunity Endotypes
Source: Immunology. 2025 Dec 9;177(4):798–809. doi: 10.1111/imm.70083 (PMC12952982; doi:10.1111/imm.70083)
Supplement: Supplementary file 1 — Table S1: Variant spike proteins, manufacturer, cell line, modifications, length and mutations. Table S2: Panel of antibodies used to screen spike protein integrity on the surface. Table S3: Mean and standard deviation of θ max values determined from the binding of aS2 antibody 40 590‐D001 to each protein channel. As this is the first time out—shall we include this? Table S4: Table of line‐of‐best fit gradients, column = row × gradient + intercept. The intercept is the limit of detection. Table S5: Table of R 2 values. Table S6: Table of medians and quartiles, broken down by exposure cohort. All values are in mg L−1. Table S7: Antibody concentrations for the Patients present in Figure 3 displayed on the upper limit of the figure. Table S8: Full results from Marascuillo Procedure analysis of Cohorts. C1 = Pre‐Pandemic, C2 = Wuhan(+), C3 = 1× vaccine, C4 = 2× vaccine and C5 = 3× vaccine. Table S9: Full endotype profile for the cohort detailing the dropout variants. Figure S1: Prevalent SARS‐CoV‐2 variants in the UK November 2021–September 2022. (A) Estimated number of cases and (B) Variant share as % of genomes sequenced from a random sample. [file IMM-177-798-s001.docx]

# Supplementary Data

Table S1 Variant Spike proteins, manufacturer, cell line, modifications, length and mutations

| Protein | Supplier | Cat # | Lot # | Type | Cell Line | AA Length | Mutations |
| --- | --- | --- | --- | --- | --- | --- | --- |
| Wuhan | Sinobiological | 40589-V08B1 | LC15OC2903 | Monomer | Baculovirus-Insect | 1209 | - |
| Alpha | Sinobiological | 40589-V08B6 | LC15MC0903 | Monomer | Baculovirus-Insect | 1206 | HV69-70 deletion, Y144 deletion, N501Y, A570D, D614G, P681H, T716I, S982A, D1118H |
| Beta | Sinobiological | 40589-V08B7 | LC15MY2607 | Monomer | Baculovirus-Insect | 1206 | L18F, D80A, D215G, LAL242-244 deletion, R246I, K417N, E484K, N501Y, D614G, A701V |
| Gamma | Sinobiological | 40589-V08B8 | LC15JU0209 | Monomer | Baculovirus-Insect | 1209 | L18F, T20N, P26S, D138Y, R190S, K417T, E484K, N501Y, D614G, H655Y, T1027I |
| Delta | Sinobiological | 40589-V08B16 | LC15OC2012 | Monomer | Baculovirus-Insect | 1207 | T19R, G142D, E156G, HR157-158 deletion, L452R, T478K, D614G, P681R, D950N |
| Omicron | Sinobiological | 40589-V08B33 | MF15DE2961 | Monomer | Baculovirus-Insect | 1206 | A67V, HV69-70del, T95I, G142D, VYY143-145del, N211del, L212I, ins214EPE, G339D, S371L, S373P, S375F, K417N, N440K, G446S, S477N, T478K, E484A, Q493R, G496S, Q498R, N501Y, Y505H, T547K, D614G, H655Y, N679K, P681H, N764K, D796Y, N856K, Q954H, N969K, L981F |
| Omicron BA.1 | Sinobiological | 40589-V08H26 | LC16JA1515 | Trimer | HEK293 | 1228 | A67V, HV69-70 deletion, T95I, G142D, VYY143-145 deletion, N211 deletion, L212I, ins214EPE, G339D, S371L, S373P, S375F, K417N, N440K, G446S, S477N, T478K, E484A, Q493R, G496S, Q498R, N501Y, Y505H, T547K, D614G, H655Y, N679K, P681H, N764K, D796Y, F817P, N856K, A892P, A899P, A942P, Q954H, N969K, L981F, K986P, V987P and furin cleavage site mutants |
| BA.2.12.1 | Sinobiological | 40589-V08H34 | MB16JU1681 | Trimer | HEK293 | 1228 | T19I, L24S, del25-27, G142D, V213G, G339D, S371F, S373P, S375F, T376A, D405N, R408S, K417N, N440K, L452Q, S477N, T478K, E484A, Q493R, Q498R, N501Y, Y505H, D614G, H655Y, N679K, P681H, S704L, N764K, D796Y, A892P, A899P, A942P, Q954H, N969K, K986P, V987P and furin cleavage site mutants |
| BA.2.75 | Acro Biosystems | SPN-C522f | 6764A-227DF1-146 | Trimer | HEK293 | 1197 | .T19I, LPP24-26del, A27S, G142D, K147E, W152R, F157L, I210V, V213G, G257S, G339H, S371F, S373P, S375F, T376A, D405N, R408S, K417N, N440K, G446S, N460K, S477N, T478K, E484A, Q498R, N501Y, Y505H, D614G, H655Y, N679K, P681H, N764K, D796Y, Q954H, N969K, R683A, R685A, F817P, A892P, A899P, A942P, K986P, V987P. Proline substitutions (F817P, A892P, A899P, A942P, K986P, V987P) and alanine substitutions (R683A and R685A) are introduced to stabilize the trimeric prefusion state of SARS-CoV-2 S protein and abolish the furin cleavage site, respectively. |
| BA.4 | Sinobiological | 40589-V08H32 | MB16JU1382 | Trimer | HEK293 | 1226 | F817P, A892P, A899P, A942P, K986P, V987P, T19I, L24del, P25del, P26del, A27S, H69del, V70del, G142D, V213G, G339D, S371F, S373P, S375F, T376A, D405N, R408S, K417N, N440K, L452R, S477N, T478K, E484A, F486V, Q498R, N501Y, Y505H, D614G, H655Y, N679K, P681H, N764K, D796Y, Q954H, N969K and furin cleavage site mutants |
| BA.5 | Sinobiological | 40589-V08H33 | MB16JU1383 | Trimer | HEK293 | 1226 | F817P, A892P, A899P, A942P, K986P, V987P, T19I, L24del, P25del, P26del, A27S, H69del, V70del, G142D, V213G, G339D, S371F, S373P, S375F, T376A, D405N, R408S, K417N, N440K, L452R, S477N, T478K, E484A, F486V, Q498R, N501Y, Y505H, D614G, H655Y, N679K, P681H, N764K, D796Y, Q954H, N969K and furin cleavage site mutants |

Table S2 Panel of Antibodies used to screen Spike protein integrity on the surface

| Cat# | Supplier | Lot # (s) | Clonality | Type | Specificity |
| --- | --- | --- | --- | --- | --- |
| RM8671 | NIST | 14HB-D-002 | Mono | Human Chimeric | RSV-F |
| 40590-D001 | Sinobiological | HA14AP2901 | Mono | Human Chimeric | S2 |
| CR3022  MAB12422-500 | Native Antigen | 21032613 | Mono | Human Chimeric | RBD |
| S1N-M122 | Acro Biosystems | C552P1-20CRF1-X3 | Mono | Human Chimeric | RBD |
| 40150-R007 | Sinobiological | HA14AP3001-B | Mono | Rabbit | RBD |
| 40592-R001 | Sinobiological |  | Mono | Rabbit | RBD |
| 40592-R190 | Sinobiological |  | Mono | Rabbit | RBD |
| 40592-R0004 | Sinobiological |  | Mono | Rabbit | RBD |
| anti S2 1034617 (MAB10557) | R&D Systems | CNEF0121021 | Mono | Mouse Ig2a | S2 |
| hu2B3E5 (ZHU1076) | Sigma Aldrich | Q3506110 | Mono | Chimeric | RBD |
|  |  |  |  |  |  |
| 40590-T62 | Sinobiological | HD15JU0103 | Poly | Rabbit | S2 |

Table S3 Mean and Standard Deviation of θmax values determined from the binding of aS2 antibody 40590-D001 to each protein channel. As this is the first time out – shall we include this??

| Protein Channel | <θ_max_> / mRIU | SD(θ_max_) / mRIU |
| --- | --- | --- |
| Protein A/G | 8.03 | 0.08 |
| Wuhan | 1.70 | 0.04 |
| Alpha | 1.05 | 0.01 |
| Beta | 0.75 | 0.04 |
| Gamma | 1.00 | 0.00 |
| Delta | 1.81 | 0.04 |
| Omicron | 0.96 | 0.01 |


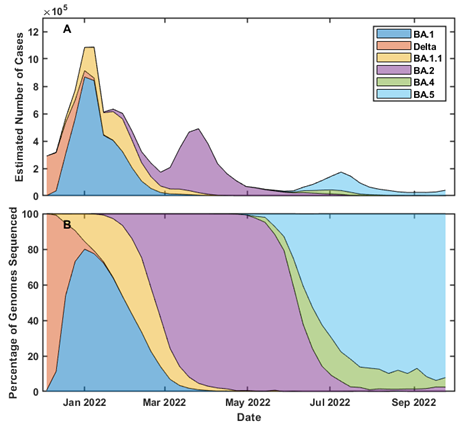


Figure S1 Prevalent SARS-CoV-2 variants in the UK November 2021-September 2022. A) Estimated number of cases and B) Variant share as % of genomes sequenced from a random sample.

Data is provided by the Wellcome Sanger Institute ^46^

Table S4 Table of Line-of-best fit gradients, column = row × gradient +intercept. The intercept is the Limit of Detection.

| **Wuhan (+)** |  | **α** | **β** | **γ** | **δ** | **Wuhan** | **BA.1** | **BA.2.12.1** | **BA.2.75** | **BA.4** | **BA.5** |
| --- | --- | --- | --- | --- | --- | --- | --- | --- | --- | --- | --- |
| α |  | 1.00 | 0.42 | 0.39 | 0.30 | 0.21 | 0.07 | 0.05 | 0.04 | 0.06 | 0.05 |
| β |  | 2.30 | 1.00 | 0.91 | 0.71 | 0.50 | 0.17 | 0.12 | 0.10 | 0.13 | 0.12 |
| γ |  | 2.51 | 1.08 | 1.00 | 0.79 | 0.56 | 0.19 | 0.15 | 0.12 | 0.16 | 0.15 |
| δ |  | 3.04 | 1.31 | 1.23 | 1.00 | 0.73 | 0.26 | 0.23 | 0.19 | 0.24 | 0.23 |
| Wuhan |  | 3.98 | 1.72 | 1.64 | 1.35 | 1.00 | 0.37 | 0.35 | 0.28 | 0.35 | 0.35 |
| BA.1 |  | 9.09 | 4.02 | 3.89 | 3.32 | 2.51 | 1.00 | 1.05 | 0.86 | 1.01 | 1.04 |
| BA.2.12.1 |  | 4.52 | 2.06 | 2.16 | 2.09 | 1.70 | 0.75 | 1.00 | 0.83 | 0.91 | 0.98 |
| BA.2.75 |  | 5.07 | 2.32 | 2.47 | 2.42 | 1.99 | 0.88 | 1.19 | 1.00 | 1.09 | 1.17 |
| BA.4 |  | 5.93 | 2.62 | 2.70 | 2.51 | 2.01 | 0.85 | 1.07 | 0.89 | 1.00 | 1.06 |
| BA.5 |  | 4.90 | 2.19 | 2.30 | 2.20 | 1.78 | 0.77 | 1.02 | 0.85 | 0.94 | 1.00 |
| **Double Vaccinated** |  |  |  |  |  |  |  |  |  |  |  |
| α |  | 1.00 | 0.64 | 0.14 | 0.14 | 0.24 | 0.14 | 0.11 | 0.13 | 0.11 | 0.11 |
| β |  | 1.11 | 1.00 | 0.18 | 0.13 | 0.25 | 0.13 | 0.06 | 0.08 | 0.06 | 0.07 |
| γ |  | 2.03 | 1.42 | 1.00 | 0.67 | 0.76 | 0.79 | 0.90 | 0.90 | 0.94 | 0.89 |
| δ |  | 3.30 | 1.83 | 1.12 | 1.00 | 1.26 | 1.15 | 1.38 | 1.40 | 1.39 | 1.32 |
| Wuhan |  | 2.87 | 1.74 | 0.65 | 0.64 | 1.00 | 0.81 | 1.00 | 1.07 | 1.01 | 0.97 |
| BA.1 |  | 1.72 | 0.94 | 0.71 | 0.61 | 0.85 | 1.00 | 1.38 | 1.38 | 1.35 | 1.30 |
| BA.2.12.1 |  | 0.61 | 0.20 | 0.36 | 0.33 | 0.47 | 0.62 | 1.00 | 1.00 | 0.98 | 0.94 |
| BA.2.75 |  | 0.72 | 0.26 | 0.34 | 0.32 | 0.48 | 0.59 | 0.95 | 1.00 | 0.95 | 0.92 |
| BA.4 |  | 0.62 | 0.19 | 0.37 | 0.33 | 0.47 | 0.60 | 0.98 | 1.00 | 1.00 | 0.95 |
| BA.5 |  | 0.68 | 0.25 | 0.39 | 0.35 | 0.50 | 0.64 | 1.03 | 1.06 | 1.04 | 1.00 |
| **Triple Vaccinated** |  |  |  |  |  |  |  |  |  |  |  |
| α |  | 1.00 | 0.57 | 0.67 | 0.45 | 0.50 | 0.49 | 0.48 | 0.48 | 0.46 | 0.45 |
| β |  | 1.11 | 1.00 | 0.79 | 0.56 | 0.61 | 0.59 | 0.56 | 0.56 | 0.53 | 0.52 |
| γ |  | 1.27 | 0.77 | 1.00 | 0.73 | 0.79 | 0.83 | 0.81 | 0.80 | 0.76 | 0.75 |
| δ |  | 1.46 | 0.91 | 1.24 | 1.00 | 1.08 | 1.16 | 1.15 | 1.12 | 1.06 | 1.06 |
| Wuhan |  | 1.32 | 0.82 | 1.10 | 0.89 | 1.00 | 1.04 | 1.06 | 1.05 | 1.00 | 0.98 |
| BA.1 |  | 1.14 | 0.69 | 1.00 | 0.83 | 0.91 | 1.00 | 1.02 | 0.99 | 0.94 | 0.94 |
| BA.2.12.1 |  | 1.00 | 0.59 | 0.89 | 0.75 | 0.84 | 0.92 | 1.00 | 0.97 | 0.94 | 0.92 |
| BA.2.75 |  | 1.02 | 0.61 | 0.90 | 0.75 | 0.85 | 0.92 | 1.00 | 1.00 | 0.96 | 0.92 |
| BA.4 |  | 1.06 | 0.62 | 0.92 | 0.76 | 0.86 | 0.94 | 1.04 | 1.03 | 1.00 | 0.96 |
| BA.5 |  | 1.09 | 0.65 | 0.97 | 0.81 | 0.91 | 1.00 | 1.08 | 1.06 | 1.03 | 1.00 |

Table S5 Table of R^2^ values

| **Wuhan (+)** |  | **α** | **β** | **γ** | **δ** | **Wuhan** | **BA.1** | **BA.2.12.1** | **BA.2.75** | **BA.4** | **BA.5** |
| --- | --- | --- | --- | --- | --- | --- | --- | --- | --- | --- | --- |
| α |  | 1.00 | 0.99 | 0.99 | 0.96 | 0.92 | 0.80 | 0.47 | 0.44 | 0.57 | 0.50 |
| β |  | 0.99 | 1.00 | 0.99 | 0.96 | 0.92 | 0.83 | 0.50 | 0.47 | 0.59 | 0.52 |
| γ |  | 0.99 | 0.99 | 1.00 | 0.98 | 0.96 | 0.87 | 0.57 | 0.55 | 0.66 | 0.60 |
| δ |  | 0.96 | 0.96 | 0.98 | 1.00 | 0.99 | 0.93 | 0.69 | 0.67 | 0.77 | 0.71 |
| Wuhan |  | 0.92 | 0.92 | 0.96 | 0.99 | 1.00 | 0.96 | 0.77 | 0.75 | 0.84 | 0.79 |
| BA.1 |  | 0.80 | 0.83 | 0.87 | 0.93 | 0.96 | 1.00 | 0.89 | 0.87 | 0.92 | 0.89 |
| BA.2.12.1 |  | 0.47 | 0.50 | 0.57 | 0.69 | 0.77 | 0.89 | 1.00 | 1.00 | 0.99 | 1.00 |
| BA.2.75 |  | 0.44 | 0.47 | 0.55 | 0.67 | 0.75 | 0.87 | 1.00 | 1.00 | 0.99 | 0.99 |
| BA.4 |  | 0.57 | 0.59 | 0.66 | 0.77 | 0.84 | 0.92 | 0.99 | 0.99 | 1.00 | 1.00 |
| BA.5 |  | 0.50 | 0.52 | 0.60 | 0.71 | 0.79 | 0.89 | 1.00 | 0.99 | 1.00 | 1.00 |
| **Double Vaccinated** |  |  |  |  |  |  |  |  |  |  |  |
| α |  | 1.00 | 0.84 | 0.54 | 0.68 | 0.82 | 0.48 | 0.26 | 0.31 | 0.26 | 0.27 |
| β |  | 0.84 | 1.00 | 0.50 | 0.50 | 0.66 | 0.35 | 0.11 | 0.15 | 0.11 | 0.13 |
| γ |  | 0.54 | 0.50 | 1.00 | 0.86 | 0.70 | 0.75 | 0.57 | 0.56 | 0.59 | 0.59 |
| δ |  | 0.68 | 0.50 | 0.86 | 1.00 | 0.90 | 0.84 | 0.67 | 0.67 | 0.68 | 0.68 |
| Wuhan |  | 0.82 | 0.66 | 0.70 | 0.90 | 1.00 | 0.83 | 0.68 | 0.72 | 0.69 | 0.69 |
| BA.1 |  | 0.48 | 0.35 | 0.75 | 0.84 | 0.83 | 1.00 | 0.92 | 0.90 | 0.90 | 0.91 |
| BA.2.12.1 |  | 0.26 | 0.11 | 0.57 | 0.67 | 0.68 | 0.92 | 1.00 | 0.97 | 0.98 | 0.98 |
| BA.2.75 |  | 0.31 | 0.15 | 0.56 | 0.67 | 0.72 | 0.90 | 0.97 | 1.00 | 0.98 | 0.98 |
| BA.4 |  | 0.26 | 0.11 | 0.59 | 0.68 | 0.69 | 0.90 | 0.98 | 0.98 | 1.00 | 1.00 |
| BA.5 |  | 0.27 | 0.13 | 0.59 | 0.68 | 0.69 | 0.91 | 0.98 | 0.98 | 1.00 | 1.00 |
| **Triple Vaccinated** |  |  |  |  |  |  |  |  |  |  |  |
| α |  | 1.00 | 0.79 | 0.92 | 0.81 | 0.81 | 0.75 | 0.69 | 0.70 | 0.70 | 0.70 |
| β |  | 0.79 | 1.00 | 0.78 | 0.71 | 0.71 | 0.64 | 0.57 | 0.58 | 0.57 | 0.58 |
| γ |  | 0.92 | 0.78 | 1.00 | 0.96 | 0.94 | 0.91 | 0.85 | 0.85 | 0.84 | 0.86 |
| δ |  | 0.81 | 0.71 | 0.96 | 1.00 | 0.98 | 0.98 | 0.93 | 0.92 | 0.90 | 0.93 |
| Wuhan |  | 0.81 | 0.71 | 0.94 | 0.98 | 1.00 | 0.97 | 0.94 | 0.95 | 0.93 | 0.94 |
| BA.1 |  | 0.75 | 0.64 | 0.91 | 0.98 | 0.97 | 1.00 | 0.97 | 0.96 | 0.94 | 0.97 |
| BA.2.12.1 |  | 0.69 | 0.57 | 0.85 | 0.93 | 0.94 | 0.97 | 1.00 | 0.98 | 0.99 | 1.00 |
| BA.2.75 |  | 0.70 | 0.58 | 0.85 | 0.92 | 0.95 | 0.96 | 0.98 | 1.00 | 0.99 | 0.99 |
| BA.4 |  | 0.70 | 0.57 | 0.84 | 0.90 | 0.93 | 0.94 | 0.99 | 0.99 | 1.00 | 0.99 |
| BA.5 |  | 0.70 | 0.58 | 0.86 | 0.93 | 0.94 | 0.97 | 1.00 | 0.99 | 0.99 | 1.00 |

Table S6 Table of medians and quartiles, broken down by exposure cohort. All values are in mg L^-1^.

| **Pre-Pandemic** |  | **α** | **β** | **γ** | **δ** | **Wuhan** | **BA.1** | **BA.2.12.1** | **BA.2.75** | **BA.4** | **BA.5** |
| --- | --- | --- | --- | --- | --- | --- | --- | --- | --- | --- | --- |
| Lower Quartile |  | 0.2 | 0.2 | 0.2 | 0.2 | 0.2 | 0.2 | 0.2 | 0.2 | 0.2 | 0.2 |
| Median |  | 0.2 | 0.2 | 0.2 | 0.2 | 0.2 | 0.4 | 0.2 | 0.2 | 0.3 | 0.3 |
| Upper Quartile |  | 0.6 | 0.2 | 0.2 | 0.2 | 0.2 | 0.7 | 1.0 | 0.7 | 0.9 | 0.9 |
|  |  |  |  |  |  |  |  |  |  |  |  |
| **Wuhan (+)** |  |  |  |  |  |  |  |  |  |  |  |
| Lower Quartile |  | 0.2 | 0.2 | 0.2 | 0.2 | 0.2 | 0.8 | 0.2 | 0.2 | 0.2 | 0.2 |
| Median |  | 0.2 | 0.2 | 0.2 | 1.4 | 1.0 | 1.0 | 1.5 | 1.2 | 1.4 | 1.3 |
| Upper Quartile |  | 2.1 | 3.8 | 2.6 | 3.6 | 1.8 | 2.0 | 2.2 | 2.0 | 2.3 | 2.2 |
|  |  |  |  |  |  |  |  |  |  |  |  |
| **Double Vaccinated** |  |  |  |  |  |  |  |  |  |  |  |
| Lower Quartile |  | 0.2 | 0.2 | 0.2 | 0.2 | 0.6 | 0.8 | 1.4 | 0.7 | 0.5 | 0.7 |
| Median |  | 0.2 | 3.4 | 1.2 | 1.7 | 1.9 | 1.8 | 3.2 | 2.6 | 2.8 | 2.8 |
| Upper Quartile |  | 5.6 | 7.5 | 3.7 | 3.0 | 3.6 | 3.6 | 6.5 | 6.4 | 6.7 | 5.8 |
|  |  |  |  |  |  |  |  |  |  |  |  |
| **Triple Vaccinated** |  |  |  |  |  |  |  |  |  |  |  |
| Lower Quartile |  | 0.2 | 0.2 | 1.5 | 0.8 | 2.1 | 2.4 | 4.8 | 3.5 | 4.4 | 3.7 |
| Median |  | 7.9 | 5.8 | 6.9 | 5.5 | 6.9 | 6.8 | 9.4 | 9.6 | 9.0 | 7.9 |
| Upper Quartile |  | 19.5 | 14.2 | 16.9 | 10.0 | 14.6 | 12.1 | 19.5 | 18.9 | 18.8 | 17.4 |

Table S7 Antibody concentrations for the Patients present in Figure 3 displayed on the upper limit of the figure.

|  | α | β | γ | δ | W | 1 | 2.12.1 | 2.75 | 4 | 5 |
| --- | --- | --- | --- | --- | --- | --- | --- | --- | --- | --- |
| W(+) | 59.6 | 32.5 | 39.2 | 45.6 | 40.5 | 21.1 | 32.3 | 26.9 | 28.2 | 40.0 |
|  | 18.7 |  | 11.2 | 16.5 | 14.6 |  |  |  |  |  |
|  | 320 | 136.1 | 123.2 | 93.2 | 64.4 | 21.3 | 12.6 | 9.7 | 15.2 | 13.5 |
|  | 12.5 | 19.8 | 12.3 | 11.7 |  |  |  |  |  |  |
|  |  | 14.1 |  |  |  |  |  |  |  |  |
|  | 9.3 |  |  |  |  |  |  |  |  |  |
| Double | 28.1 | 19.7 |  |  |  |  |  |  |  |  |
| Vaccinated | 52.8 | 50.9 |  |  |  |  |  |  |  |  |
|  |  |  |  |  |  |  | 27.5 | 26.9 | 26.2 | 25.1 |
|  | 74.2 | 40.2 |  |  |  |  |  |  |  |  |
|  |  | 29.5 |  |  |  |  |  |  |  |  |
|  | 18.9 |  |  |  |  | 20.7 | 29.4 | 27.3 | 29.8 | 27.8 |
|  |  | 18.6 |  |  |  |  |  |  |  |  |
| Triple | 61.4 | 46.7 | 61.2 | 54.8 | 57.9 | 64.6 | 64.3 | 59.0 | 56.4 | 59.5 |
| Vaccinated | 80.7 | 44.2 | 44.1 |  |  |  |  |  |  |  |
|  |  | 42.1 |  |  |  |  |  |  |  |  |
|  | 51.5 |  |  |  |  |  |  |  |  |  |

Table S8. Full results from Marascuillo Procedure analysis of Cohorts. C1 = Pre-Pandemic, C2 = Wuhan(+), C3 = 1x Vaccine, C4 = 2x Vaccine and C5 = 3x Vaccine.

| Endotype | Cohorts (labelled 1-5) |  | Value | Critical Range | Significant? |
| --- | --- | --- | --- | --- | --- |
| U(+) | C1 vs C2 |  | 0.111 | 0.161 | No |
| U(+) | C1 vs C3 |  | 0.000 | 0.000 | No |
| U(+) | C1 vs C4 |  | 0.220 | 0.199 | Yes |
| U(+) | C1 vs C5 |  | 0.585 | 0.237 | Yes |
| U(+) | C2 vs C3 |  | 0.111 | 0.161 | No |
| U(+) | C2 vs C4 |  | 0.108 | 0.256 | No |
| U(+) | C2 vs C5 |  | 0.474 | 0.287 | Yes |
| U(+) | C3 vs C4 |  | 0.220 | 0.199 | Yes |
| U(+) | C3 vs C5 |  | 0.585 | 0.237 | Yes |
| U(+) | C4 vs V5 |  | 0.366 | 0.310 | Yes |
| U(-) | C1 vs C2 |  | 0.507 | 0.382 | Yes |
| U(-) | C1 vs C3 |  | 0.505 | 0.496 | Yes |
| U(-) | C1 vs C4 |  | 0.715 | 0.333 | Yes |
| U(-) | C1 vs C5 |  | 0.788 | 0.310 | Yes |
| U(-) | C2 vs C3 |  | 0.002 | 0.460 | No |
| U(-) | C2 vs C4 |  | 0.208 | 0.276 | No |
| U(-) | C2 vs C5 |  | 0.281 | 0.248 | Yes |
| U(-) | C3 vs C4 |  | 0.210 | 0.419 | No |
| U(-) | C3 vs C5 |  | 0.283 | 0.401 | No |
| U(-) | C4 vs V5 |  | 0.073 | 0.161 | No |
| U(±) | C1 vs C2 |  | 0.396 | 0.393 | Yes |
| U(±) | C1 vs C3 |  | 0.505 | 0.496 | Yes |
| U(±) | C1 vs C4 |  | 0.495 | 0.375 | Yes |
| U(±) | C1 vs C5 |  | 0.203 | 0.381 | No |
| U(±) | C2 vs C3 |  | 0.109 | 0.469 | No |
| U(±) | C2 vs C4 |  | 0.100 | 0.338 | No |
| U(±) | C2 vs C5 |  | 0.345 | 0.345 | No |
| U(±) | C3 vs C4 |  | 0.453 | 0.453 | No |
| U(±) | C3 vs C5 |  | 0.459 | 0.459 | No |
| U(±) | C4 vs V5 |  | 0.324 | 0.324 | No |

Table S9. Full Endotype Profile for the cohort detailing the dropout variants

| Endotype | Overall Incidence (%)  (*n*=148) | Endotype Incidence (%) By Cohort | | | | | | | | | | | | | | |
| --- | --- | --- | --- | --- | --- | --- | --- | --- | --- | --- | --- | --- | --- | --- | --- | --- |
|  |  | Pre-pan  (16) | W (+)  (36) | **1x Vaccine**  **(13)** | | **2x Vaccine**  **(41)** | | | **3x Vaccine**  **(41)** | | | | | | | **Un**  **(1)** |
|  |  |  |  | AZ (9) | Pf (4) | AZ (23) | Pf (17) | Un (1) | AZ-AZ-Pf (12) | AZ-AZ-M (9) | Pf-Pf-Pf (10) | Pf-Pf-M (3) | M-M-M (1) | Nx-Pf (1) | Un (5) |  |
| **Summary** |  |  |  |  |  |  |  |  |  |  |  |  |  |  |  |  |
| **U(+)** | 25.7 |  | 11.1 |  |  | 26.1 | 17.6 |  | 58.3 | 88.9 | 40.0 | 33.3 | 100.0 |  | 60.0 | 100.0 |
| **Sub-Cohort** |  |  |  |  | | 22.0 | | | 59 | | | | | | |  |
| **U(-)** | 22.3 | 81.3 | 30.6 | 22.2 | 50.0 | 8.7 | 11.8 |  | 8.3 |  |  |  |  |  |  |  |
| **Sub-Cohort** |  |  |  | 30.8 | | 9.8 | | | 2.4 | | | | | | |  |
| **U(±) Any Dropout** | 52.0 | 18.8 | 58.3 | 77.8 | 50.0 | 65.2 | 70.6 | 100.0 | 33.3 | 11.1 | 60.0 | 66.7 |  | 100.0 | 40.0 |  |
| **Sub-Cohort** |  |  |  | 69.2 | | 68.3 | | | 39.0 | | | | | | |  |
|  |  |  |  |  |  |  |  |  |  |  |  |  |  |  |  |  |
| **Dropout Endotypes** **(±)** | |  |  |  |  |  |  |  |  |  |  |  |  |  |  |  |
| **Single Dropout** |  |  |  |  |  |  |  |  |  |  |  |  |  |  |  |  |
| β | 2.0 |  |  |  |  |  | 5.9 |  | 8.3 |  | 10.0 |  |  |  |  |  |
| δ | 0.7 |  |  |  |  |  |  | 100.0 |  |  |  |  |  |  |  |  |
| Total | 2.7 |  |  |  |  |  | 5.9 | 100.0 | 8.3 |  | 10.0 |  |  |  |  |  |
|  |  |  |  |  |  |  |  |  |  |  |  |  |  |  |  |  |
| **Double (-)** |  |  |  |  |  |  |  |  |  |  |  |  |  |  |  |  |
| 4/5 | 0.7 |  | 2.8 |  |  |  |  |  |  |  |  |  |  |  |  |  |
| Total | 0.7 |  | 2.8 |  |  |  |  |  |  |  |  |  |  |  |  |  |
|  |  |  |  |  |  |  |  |  |  |  |  |  |  |  |  |  |
| **Triple (-)** |  |  |  |  |  |  |  |  |  |  |  |  |  |  |  |  |
| ag/1 | 1.4 |  |  |  |  |  | 11.8 |  |  |  |  |  |  |  |  |  |
| abd | 0.7 |  |  |  |  |  |  |  |  |  |  |  |  |  | 20.0 |  |
| abg | 1.4 |  |  |  |  | 4.3 |  |  |  |  | 10.0 |  |  |  |  |  |
| W/d/1 | 0.7 |  |  |  |  |  | 5.9 |  |  |  |  |  |  |  |  |  |
| agd | 0.7 |  |  |  |  |  |  |  |  |  |  |  |  | 100.0 |  |  |
| abW | 0.7 |  |  |  |  |  | 5.9 |  |  |  |  |  |  |  |  |  |
| 2.75/4/5 | 0.7 |  |  |  |  | 4.3 |  |  |  |  |  |  |  |  |  |  |
| **Total** | 6.1 |  |  |  |  | 8.7 | 23.5 |  |  |  | 10.0 |  |  | 100.0 | 20.0 |  |
| **Quadruple(-)** |  |  |  |  |  |  |  |  |  |  |  |  |  |  |  |  |
| abg1 | 0.7 |  | 2.8 |  |  |  |  |  |  |  |  |  |  |  |  |  |
| 2.12.1/2.75/4/5 | 2.7 |  | 2.8 | 11.1 |  | 4.3 | 5.9 |  |  |  |  |  |  |  |  |  |
| abgW | 0.7 |  | 2.8 |  |  |  |  |  |  |  |  |  |  |  |  |  |
| a/b/g/d | 1.4 |  |  |  |  |  | 5.9 |  | 8.3 |  |  |  |  |  |  |  |
| **Total** | 5.4 |  | 8.3 | 11.1 |  | 4.3 | 11.8 |  | 8.3 |  |  |  |  |  |  |  |
| **Quintuple(-)** |  |  |  |  |  |  |  |  |  |  |  |  |  |  |  |  |
| a/b/g/1/W | 1.4 |  | 2.8 |  |  | 4.3 |  |  |  |  |  |  |  |  |  |  |
| a/g/d/1/W | 1.4 |  |  |  |  | 4.3 | 5.9 |  |  |  |  |  |  |  |  |  |
| a/b/d/1/W | 1.4 |  |  |  |  |  |  |  |  |  | 10.0 | 33.3 |  |  |  |  |
| a/b/g/d/1 | 0.7 |  |  |  |  |  | 5.9 |  |  |  |  |  |  |  |  |  |
| a/g/2.75/4/5 | 0.7 |  |  |  |  |  | 5.9 |  |  |  |  |  |  |  |  |  |
| 1/2.12.1/2.75/4/5 | 1.4 |  |  |  |  | 4.3 |  |  |  | 11.1 |  |  |  |  |  |  |
| a/b/g/d/W | 2.0 |  | 2.8 | 11.1 |  |  |  |  |  |  |  |  |  |  | 20.0 |  |
| **Total** | 8.8 |  | 5.6 | 11.1 |  | 13.0 | 17.6 |  |  | 11.1 | 10.0 | 33.3 |  |  | 20.0 |  |
| **6(-)** |  |  |  |  |  |  |  |  |  |  |  |  |  |  |  |  |
| 1/2.12.1/2.75/4/5/W | 1.4 |  | 2.8 | 11.1 |  |  |  |  |  |  |  |  |  |  |  |  |
| d/1/2.12.1/2.75/4/5 | 0.7 |  | 2.8 |  |  |  |  |  |  |  |  |  |  |  |  |  |
| a/2.12/2.75/4/5/W | 0.7 |  | 2.8 |  |  |  |  |  |  |  |  |  |  |  |  |  |
| a/b/g/d/1/W | 4.1 |  | 5.6 |  |  |  | 5.9 |  | 8.3 |  | 10.0 | 33.3 |  |  |  |  |
| **Total** | 6.8 |  | 13.9 | 11.1 |  |  | 5.9 |  | 8.3 |  | 10.0 | 33.3 |  |  |  |  |
| **7(-)** |  |  |  |  |  |  |  |  |  |  |  |  |  |  |  |  |
| b/g/2.75/4/5/2.12/1 | 0.7 |  | 2.8 |  |  |  |  |  |  |  |  |  |  |  |  |  |
| a/b/g/d/2.75/1/W | 2.0 |  | 2.8 |  |  | 4.3 |  |  |  |  | 10.0 |  |  |  |  |  |
| a/b/g/d/5/1/W | 0.7 |  |  |  |  | 4.3 |  |  |  |  |  |  |  |  |  |  |
| **Total** | 3.4 |  | 5.6 |  |  | 8.7 |  |  |  |  | 10.0 |  |  |  |  |  |
| **8(-)** |  |  |  |  |  |  |  |  |  |  |  |  |  |  |  |  |
| ab/2.12/2.75/4/5/1/W | 0.7 |  | 2.8 |  |  |  |  |  |  |  |  |  |  |  |  |  |
| abgd/2.12/2.75/1/W | 0.7 |  | 2.8 |  |  |  |  |  |  |  |  |  |  |  |  |  |
| abgd/2.75/5/1/W | 0.7 |  |  |  |  |  | 5.9 |  |  |  |  |  |  |  |  |  |
| abgd/2.75/4/1/W | 0.7 |  |  |  |  | 4.3 |  |  |  |  |  |  |  |  |  |  |
| abgd/2.75/4/5/W | 0.7 |  |  | 11.1 |  |  |  |  |  |  |  |  |  |  |  |  |
| ad/2.12/2.75/4/5/1/W | 0.7 |  |  |  |  | 4.3 |  |  |  |  |  |  |  |  |  |  |
| agd/2.12/2.75/4/5/1 | 0.7 |  |  |  | 25.0 |  |  |  |  |  |  |  |  |  |  |  |
| gd/2.12/2.75/4/5/1/W | 0.7 |  | 2.8 |  |  |  |  |  |  |  |  |  |  |  |  |  |
| **Total** | 5.4 |  | 8.3 | 11.1 | 25.0 | 8.7 | 5.9 |  |  |  |  |  |  |  |  |  |
| **9(-)** |  |  |  |  |  |  |  |  |  |  |  |  |  |  |  |  |
| abg/2.12/2.75/4/5/1/W | 0.7 |  | 2.8 |  |  |  |  |  |  |  |  |  |  |  |  |  |
| abgd/2.12/2.75/4/5/W | 2.0 |  | 2.8 |  |  | 4.3 |  |  |  |  | 10.0 |  |  |  |  |  |
| abgd/2.12/4/5/1/W | 0.7 |  |  |  |  | 4.3 |  |  |  |  |  |  |  |  |  |  |
| abgd/2.75/4/5/1/W | 2.7 |  |  | 22.2 | 25.0 | 4.3 |  |  |  |  |  |  |  |  |  |  |
| agd/2.12/2.75/4/5/1/W | 5.4 | 6.3 | 8.3 | 11.1 |  | 8.7 |  |  | 8.3 |  |  |  |  |  |  |  |
| bgd/2.12/2.75/4/5/1/W | 1.4 | 12.5 |  |  |  |  |  |  |  |  |  |  |  |  |  |  |
| **Total** | 12.8 | 18.8 | 13.9 | 33.3 | 25.0 | 21.7 |  |  | 8.3 |  | 10.0 |  |  |  |  |  |
